# Serum lipid levels are positively correlated with lumbar disc herniation—a retrospective study of 790 Chinese patients

**DOI:** 10.1186/s12944-016-0248-x

**Published:** 2016-04-18

**Authors:** Yuedong Zhang, Yunpeng Zhao, Mei Wang, Meng Si, Jingkun Li, Yong Hou, Jialin Jia, Lin Nie

**Affiliations:** Department of Orthopaedics, Qilu Hospital, Shandong University, Jinan, Shandong 250012 P. R. China; Department of Orthopaedics, Taian Central Hospital, Taian, Shandong 271000 P. R. China; College of Information Engineering, Taishan Medical University, Taian, Shandong 271016 P. R. China; Medical College of Shandong University, Jinan, Shandong 250012 P. R. China

**Keywords:** Serum lipids, Intervertebral disc degeneration, Cholesterol, Triglycerides, LDL-C, HDL-C

## Abstract

**Background:**

Abnormal serum lipid levels have been shown to be associated with the occurrence of atherosclerosis, but little is known about the relationships of them with the risk of developing intervertebral disc degeneration (IVDD) in Chinese population.

**Methods:**

We performed a case–control study to assess the relationship between serum lipid levels and lumbar disc degeneration. A total of 790 Chinese patients were recruited for this study at the time of hospitalization. We examined fasting serum lipid levels of total cholesterol (TC), triglycerides (TG), low-density lipoprotein cholesterol (LDL-C) and high-density lipoprotein cholesterol (HDL-C). 396 patients (235 men and 161 women; mean age: 41.07 years) underwent surgery for single-level lumbar disc herniation. A control group of 394 patients (225 men and 169 women; mean age: 42.1 years) underwent surgery for wounded lower limbs during the same period. Patients in the control group were collected randomly from among patients who were age- and sex-matched patients with the case group.

**Results:**

Patients with lumbar disc herniation had significantly higher TC and LDL-C serum concentrations (*P* < 0.001 for both) than controls. Percentage of High-TC, High-TG, High-LDL-C, borderline High-TC and borderline High-LDL-C were significantly higher in the disc herniation group (*P* = 0.017, *P* = 0.002, *P* = 0.039, *P* =0.002 and *P* < 0.001, respectively). Ratios of TC/HDL-C and LDL-C/HDL-C were significantly associated with disc herniation (P < 0.001 for both). Logistic regression revealed that patients with higher serum LDL-C levels had a higher risk of disc herniation, in which odds ratio (OR) was 1.462 and confidence interval (CI) was 1.179 ~ 1.813. Moreover, patients with High-TG and borderline High-LDL-C had a higher probability of disc herniation (OR: 2.974, CI: 1.488 ~ 5.945, statistical power: 100 %; OR: 1.626, CI: 1.012 ~ 2.612, statistical power: 61.4 %, respectively). However, hyperlipidaemia did not seem to be associated with the herniated segment of the lumbar intervertebral disc (*p* = 0.374).

**Conclusions:**

The present study suggests that dyslipidaemia may be associated with a higher risk of developing lumbar disc herniation. Serum lipid levels could be a useful predictor for intervertebral disc degeneration in Chinese population.

## Background

Low back pain (LBP) is a major public health problem that causes individual suffering and economic loss. Moreover, 70 to 85 % of people suffer from low back pain at some time during their lifetime [1]. Sciatica, which is characterized by low back pain with radiculopathy, is a common clinical symptom that affects approximately 40 % of the adult population at some time [2], often has a lengthy course and causes long-term disability [3]. Lumbar disc herniation (LDH) is a major cause of low back pain and sciatica. Lumbar disc abnormalities are frequently found in asymptomatic individuals by magnetic resonance imaging [4]. However, only 4 % to 6 % of the population presents with symptomatic sciatica [2]. Though many scholars have investigated the aetiology and treatment of intervertebral disc degeneration (IVDD) [5–8], the underlying pathophysiologic mechanism remains unclear. Furthermore, abnormal serum lipid levels are well-known risk factors for the development of atherosclerosis [9, 10]. We therefore asked whether there was an association between serum lipid levels and IVDD.

In 2007, the Joint Committee for Developing Chinese Guidelines on Prevention and Treatment of Dyslipidemia in Adults reported that the incidence of ischemic heart disease due to atherosclerosis, such as cardiovascular diseases and ischemic stroke, increased from 1984 to 1999. Furthermore, a cohort study showed that elevated serum levels of TC or LDL-C were one of the independent risk factors for ischemic heart disease. The prevalence of dyslipidaemia in Chinese adults was 18.6 % for both genders in 2002, while rates of hypercholesterolaemia, hypertriglyceridemia and Low-HDL-C were 2.9, 11.9 and 7.4 %, respectively. Moreover, during the past few decades, the incidence of LBP has increased in China, resulting in a heightened interest in understanding its aetiology and developing effective therapeutic strategies.

To evaluate the relationship between serum lipid levels in Chinese patients and lumbar disc herniation, we undertook a cross-sectional group comparison case–control study of serum lipid levels obtained from patients with lumbar disc herniation, and compared them with a control group of patients with wounded lower limbs. In the present study, fasting serum TC, TG, LDL-C and HDL-C concentrations were measured by biochemical analyses. We further investigated the associations between incidences of dyslipidaemia, lipoprotein ratios and IVDD. We hypothesized that serum lipid levels were positively correlated with lumbar disc herniation.

## Results

### Patient population

Characteristics of the overall population are shown in Table 1. The case group included 396 subjects (235 men and 161 women) whose mean age was 41.07 years and mean body mass index was 24.21 kg/m^2^. The control group consisted of 394 subjects (225 men and 168 women) whose mean age was 42.1 years and mean body mass index was 24.58 kg/m^2^. No difference in age, gender, body mass index and labour intensity was observed between the two groups.

**Table 1:** Baseline characteristics of participants (*N* = 790)

Variables	Group 1 (disc herniation)	Group 2 (control group)	*P* value
	(*n* = 396)	(*n* = 394)	
Gender (M/F)	235/161	225/168	0.524
Age (years)	41.07 ± 11.34	42.1 ± 14.35	0.263
Range (years)	18–79	18–82	
BMI (kg/m^2^)	24.21 ± 3.62	24.58 ± 3.57	0.144
Labour intensity			0.846
Light (%)	35.1	32.5	
Moderate (%)	18.7	25.1	
Heavy (%)	46.2	42.4	

Data on age and BMI are given as the means ± SD

*BMI*, body mass index

### TC and LDL-C, but not TG or HDL-C serum levels were elevated in patients with lumbar disc herniation

In the present study, serum was collected from all patients in vacutainer tubes, and biochemical analyses were performed to determine lipid levels. As shown in Table 2, patients with lumbar disc herniation had significantly higher serum concentrations of TC 4.75 mmol/L (range, 2.03–10.27 mmol/L) (*P* < 0.001) and LDL-C 2.92 mmol/L (range, 0.68–8.33 mmol/L) (*P* < 0.001) compared with patients in the control group, TC 4.41 mmol/L (range, 1.69–7.35 mmol/L) and LDL-C 2.62 mmol/L (range, 0.53–4.99 mmol/L). However, there was no association between serum levels of TG or HDL-C and lumbar disc herniation between the two groups, suggesting that, in these cases, TG or HDL-C are not involved in the pathogenesis of disc herniation.

**Table 2:** The concentrations of serum lipids in two groups (mmol/L)

Serum lipids	Disc herniation	Control group	*t* value	*P* value
	(*n* = 396)	(*n* = 394)		
TC	4.75 ± 1.02	4.41 ± 0.90	4.984	<0.001
TG	1.46 ± 0.89	1.35 ± 0.94	1.669	0.096
LDL-C	2.92 ± 0.86	2.62 ± 0.73	5.129	<0.001
HDL-C	1.32 ± 0.30	1.34 ± 0.47	0.641	0.521

Data are given as the means ± SD, and analysed with independent-sample t-tests

### Incidence of dyslipidaemia was enhanced in patients with lumbar disc herniation

As indicated in Table 3, in the disc herniation group, the incidence of High-TC, High-TG, High-LDL-C and Low-HDLC was 7.1, 17.4, 6.8 and 15.7 %, respectively, compared to an incidence of 3.3, 9.9, 3.6 and 16 %, respectively, in the control group. The incidence of High-TC, High-TG and High-LDL-C was significantly higher in the disc herniation group compared to the control group (*P* = 0.017, *P* = 0.002 and *P* = 0.039, respectively). Among the 396 patients with disc herniation, 89 (24.3 %) had borderline High-TC, 51 (15.6 %) had borderline High-TG and 82 (22.3 %) had borderline High-LDL-C compared to 59 (15.5 %), 45 (12.7 %) and 38 (10.1 %), respectively, among the 394 control patients. The incidence of borderline High-TC and borderline High-LDL-C was significantly higher in the disc herniation group compared to the control group (*P* = 0.002 and *P* < 0.001, respectively).

**Table 3:** Incidences of dyslipidaemia in two groups

Serum lipid concentration	Disc herniation	Control group	*P* value
	*N*(%)	*N*(%)	
TC			
5.18 ~ 6.19 mmol/L	89(24.3)	59(15.5)	0.002
≥6.22 mmol/L	28(7.1)	13(3.3)	0.017
TG			
1.70 ~ 2.25 mmol/L	51(15.6)	45(12.7)	0.273
≥2.26 mmol/L	69(17.4)	39(9.9)	0.002
LDL-C			
3.37 ~ 4.12 mmol/L	82(22.3)	38(10.1)	<0.001
≥4.14 mmol/L	27(6.8)	14(3.6)	0.039
HDL-C			
<1.04 mmol/L	62(15.7)	63(16)	0.898

Categorical variables are expressed as a percentage of the number and analyzed with a chi-square test

### TC/HDL-C and LDL-C/HDL-C ratios were elevated in case group

**Fig. 1:**
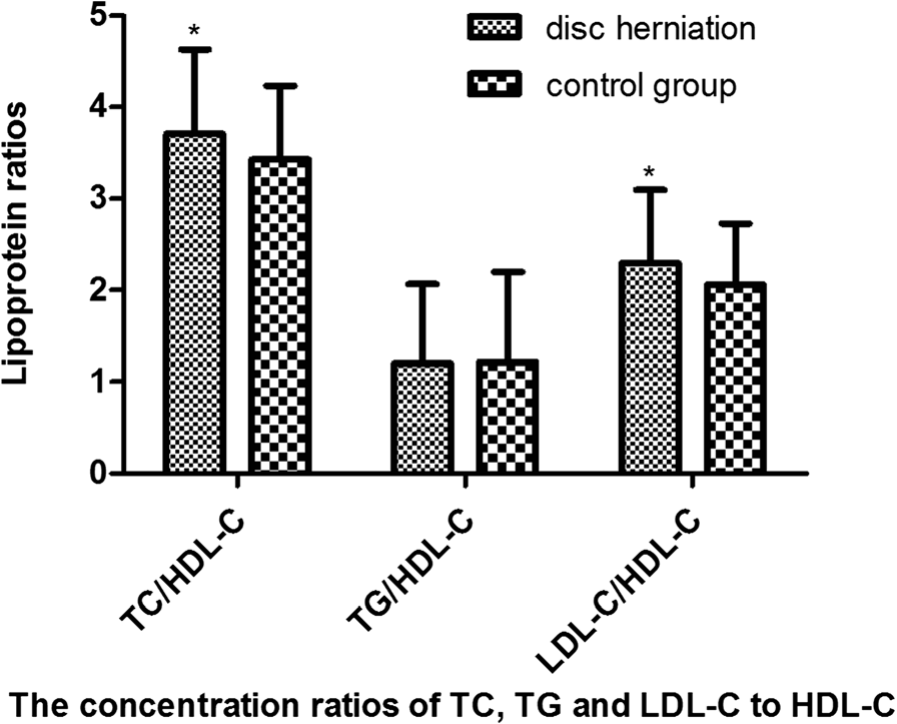
Lipoprotein ratios in two groups. The ratios of TC/HDL-C and LDL-C/HDL-C were significantly higher in the disc herniation group compared to the control group, *p < 0.001

In case group, the ratio of TC/HDL-C, TG/HDL-C and LDL-C/HDL-C was 3.71, 1.20 and 2.30, respectively, compared to a ratio of 3.43, 1.22, 2.06, respectively, in the control group. The ratios of TC/HDL-C and LDL-C/HDL-C were significantly higher in the disc herniation group compared to the control group (*P* < 0.001 for both) (Fig. 1).

### Hyperlipidaemia was negatively associated with the herniated segment of lumbar intervertebral disc

**Fig. 2:**
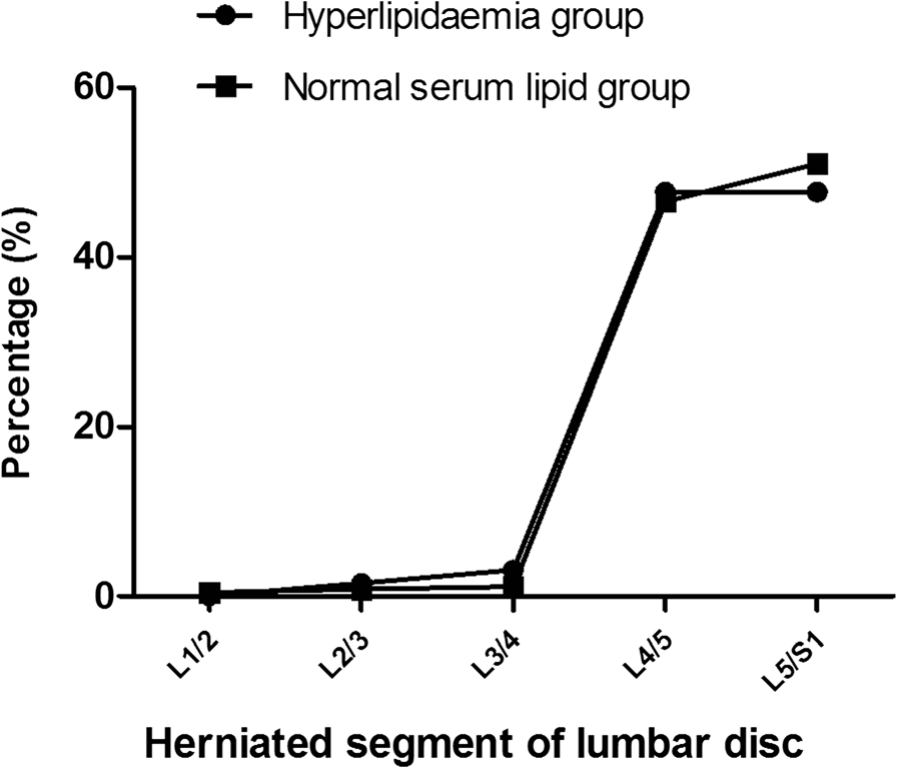
The percentage of herniated segment of lumbar intervertebral disc in hyperlipidaemia group and normal serum lipids group. There was no significant difference in herniated segment between the two groups (*p* = 0.374)

We analyzed the categorical data on patients with disc herniation to examine the relationships between hyperlipidaemia and herniated segment of lumbar disc in disc herniation group. We observed that, in hyperlipidaemia group (*n* = 130), the percentage of herniated segment of L1/2, L2/3, L3/4, L4/5 and L5/S1 was 0, 1.5, 3.1, 47.7 and 47.7, respectively, compared to a percentage of 0.4, 0.8, 1.1, 46.6 and 51.1, respectively, in the normal serum lipids group (*n* = 266). There was no significant difference in herniated segment between the two groups (*p* = 0.374) (Fig. 2). The result suggested that hyperlipidaemia did not affect the herniated segment of the lumbar intervertebral disc.

### Multivariate logistic regression of multiple covariates and the risk of intervertebral disc degeneration

As shown in Table 4, after adjusting for variables of serum lipid levels including TC, TG, HDL-C, and LDL-C (model 1), the odds ratio (OR) for having a disc herniation with an elevated LDL-C was 1.596 (CI, 1.327 ~ 1.921). After adjusting for hyperlipidaemia variables including High-TC, High-TG, High-LDL-C and Low-LDL-C (model 2) and borderline High-TC, borderline High-TG and borderline High-LDL-C (model 3), the OR for having a disc herniation with High-TC, High-TG and borderline High-LDL-C were 2.045 (CI, 1.036 ~ 4.036), 1.843 (CI, 1.207 ~ 2.815), 2.447 (CI, 1.618 ~ 3.699) respectively. In model 4, the concentration ratio of LDL-C/HDL-C was significantly associated with disc herniation (OR, 1.578; CI, 1.293 ~ 1.925). However, the associations between High-TC, LDL-C/HDL-C ratio and the risk of disc herniation became insignificant after adjusting for the variables in model 5. Finally, LDL-C levels, High-TG and borderline High-LDL-C were significantly associated with disc herniation (model 5). These results suggested that patients with higher serum LDL-C levels had a higher risk of disc herniation (OR, 1.462; CI, 1.179 ~ 1.813). Moreover, patients with High-TG and borderline High-LDL-C had a higher probability of developing disc herniation (OR: 2.974, CI: 1.488 ~ 5.945, statistical power: 100 %; OR: 1.626, CI: 1.012 ~ 2.612, statistical power: 61.4 %, respectively).

**Table 4:** Multivariate logistic regression of multiple covariates and the risk of intervertebral disc degeneration

Variables	Risk of disc herniation		
	OR	95 % CI	*P* value
Model 1			
LDL-C levels	1.596	1.327 ~ 1.921	<0.001
Model 2			
High-TC	2.045	1.036 ~ 4.036	0.039
High-TG	1.843	1.207 ~ 2.815	0.005
Model 3			
Borderline High-LDL-C	2.447	1.618 ~ 3.699	<0.001
Model 4			
LDL-C/HDL-C ratio	1.578	1.293 ~ 1.925	<0.001
Model 5			
LDL-C levels	1.462	1.179 ~ 1.813	0.001
High-TG	2.974	1.488 ~ 5.945	0.002
Borderline High-LDL-C	1.626	1.012 ~ 2.612	0.045

Model 1, adjusted for the variables of serum lipid levels including TC, TG, HDL-C, and LDL-C; Model 2, adjusted for the hyperlipidaemia variables including High-TC, High-TG, High-LDL-C and Low-LDL-C; Model 3, adjusted for the hyperlipidaemia variables including borderline High-TC, borderline High-TG and borderline High-LDL-C; Model 4, adjusted for the variables of lipoprotein ratios including TC/HDL-C, TG/HDL-C and LDL-C/HDL-C; Model 5, adjusted for the variables in model 1 plus the variables in model 2, model 3 and model 4

*OR* odds ratio, *CI* confidence interval

## Discussion

Intervertebral disc degeneration (IVDD) has been extensively studied [5–8], though the precise pathophysiologic mechanism underlying the disorder remains unclear. A synergistic effect involving many factors plays a role in the development of IVDD. These include abnormal mechanical loading [11, 12], nutrition-related metabolic disorders [13, 14], upregulation of matrix metalloproteinases (MMPs) [15, 16] and ADAMTS [17, 18] expression and activation of cytokines [19, 20]. Additional contributing factors are aging [21], physical activity [22], and heredity [23].

Previous reports have identified an association between dyslipidaemia and rotator cuff tears [24] or Achilles tendon ruptures [25]. Because these conditions share common features with intervertebral disc (IVD), such as a poor vascular supply, we were prompted to investigate whether there was a relationship between IVDD and abnormal serum lipid levels. We therefore designed a case–control study to determine the serum lipid levels in patients with lumbar disc herniation.

Recently, regional investigations have suggested a potential association between serum lipids and degenerative IVD disease or low back pain. A correlation between higher total cholesterol (TC), LDL cholesterol, triglycerides (TG) levels and sciatica in men has been identified in Finnish patients [3]. A large cohort study of male office-based civil servants in London found that after adjusting for multiple factors, triglycerides (TG) were associated with sick-day related absences due to back pain [26]. A follow-up study reported that high total cholesterol (TC) and triglycerides (TG) in a working population predicted the incidence of radiating low back pain [27]. High LDL cholesterol was also associated with disc degeneration in the elderly [28]. A case–control study concluded that patients with symptomatic herniated lumbar disc had higher triglyceride (TG) and total cholesterol (TC) serum concentrations [29], though there was no information collected about LDL-C and HDL-C levels.

However, none of above studies on outcomes have evaluated the association of the borderline hyperlipidaemia and lipoprotein ratios with IVDD. In the current study, we analyzed the serum concentrations of TC, TG, LDH-C and LDL-C, incidences of dyslipidaemia (hyperlipidaemia and borderline hyperlipidaemia) and lipoprotein ratios in two groups. After adjusting for hyperlipidaemia variables (Table 4), we found that adult patients (male and female) with lumbar disc herniation had higher LDL-C serum concentration and incidence of High-TG or borderline High-LDL-C compared to those without disc herniation, The mentioned three lipids are well accepted for the detrimental role in atherosclerosis, which may destruct the blood supply of IVD and lead to LDH, but there might be some other potential pathways which are required for further investiagation. On the basis of our observation, the key finding of this study was that LDL-C, High-TG and borderline High-LDL-C could be better predictors for the occurrence and development of IVDD than other parameters. Serum lipid levels may therefore represent a risk factor for IVDD pathology.

The precise pathophysiologic mechanism underlying the connection between serum lipid levels and lumbar disc herniation remains unclear. Previous studies have suggested a potential association between lumbar disc degeneration and atherosclerosis. An autopsy of 86 men study concluded that atherosclerosis in the abdominal aorta and especially stenosis of the ostia of segmental arteries may play a potential role in lumbar disc degeneration [30]. In a 25-year follow-up study in Framingham, the authors reported that calcific atherosclerotic deposits in the posterior wall of the abdominal aorta increased the risk for the development of disc degeneration and were associated with back pain [31].

In the current study, our results suggest that dyslipidaemia may be associated with a higher risk of developing lumbar disc herniation. Thus, there might be a link that connects serum lipid levels and LDH. One logical link between them is atherosclerosis, which could be responsible for a decreased in the blood supply to the corresponding lumbar segment and causes the malnutrition of IVD. The insufficient nutrient supply to IVD cells ultimately leads to IVDD disease [32]. Theoretically, the lumbar vertebral bodies are supplied by the branches of lumbar arteries, which originate from the lowest part of the abdominal aorta. However, the lowest part often shows the earliest lesions in the process of atherosclerosis. Thus, atheromatous plaques and calcification tend to form in or around ostia of the branches of lumbar arteries, resulting in stenosis or obliteration of the segmental lumbar arteries [31, 33], which obstructs nutrient supply to a corresponding lumbar segment and leads to IVDD.

The IVD is a poorly vascularized whose primary nutritional supply is *via* blood capillary penetration of the vertebral bodies through to the endplate [34]. High serum cholesterol [35] and triglycerides [9, 10] levels are established risk factors for atherosclerosis. Furthermore, dyslipidaemia can also accelerate the atherosclerosis process and its morbid consequences [36], which will destruct the vascular suppy to the already poor vascularized IVD.

**Fig. 3:**
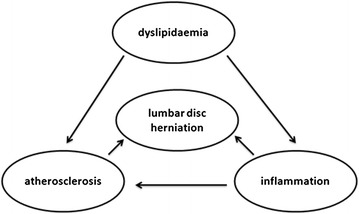
Relationships between dyslipidaemia, atherosclerosis, inflammation, and lumbar disc herniation. The potential pathogenetic mechanism underlining the connection between serum lipid levels and lumbar disc herniation might be through atherosclerosis and inflammatory pathways

Another potential pathogenetic mechanism underlying the link between serum lipid levels and LDH could be through inflammatory pathways. Previous studies reported that pro-inflammatory cytokine were associationed with serum lipid levels [37, 38]. Activation of cytokines plays a role in the development of disc degeneration [19, 20, 39]. Besides, it is possible that increased serum lipid levels enhance inflammatory response or basic level of systemic inflammation, resulting in disc degeneration [27]. Moreover, atherosclerosis as an inflammatory disease [40] is initiated by endothelial injury due to oxidative stress in the context of dyslipidaemia [41]. The relationship between dyslipidaemia, atherosclerosis, inflammation, and disc degeneration is depicted in Fig. 3.

As is known, elevated levels of TC, TG or LDL-C and reduced HDL-C level are atherogenic lipid marker. An elevated level of LDL-C is a major risk factor for the development of atherosclerosis [42]. The management of cardiovascular disease has traditionally focused on reducing LDL-C or total lipid levels [43], In this study, we found that LDL-C levels were significantly correlated with lumbar disc degeneration (LDH) and High-TG or borderline High-LDL-C predicted a higher incidence of LDH. This association opens the way for a new approach to reducing the risk of intervertebral disc degeneration (IVDD) disease by controlling serum lipid levels. Clinically, there are different medicines to reduce the abnormal serum lipids, such as statins and bile acid resins. Moreover, Scicchitano P et al. [44] reported that other interventions such as nutraceuticals and functional food ingredients may also play a role in promoting healthy control of dyslipidaemia.

The present case–control study is a retrospective design,and a limitation of this design is the cross-sectional nature, which cannot completely resolve issues concerning temporality [29]. While some retrospective information can be collected from medical records, retrospective study leaves causality undetermined. we cannot determine a direct causal relationship nor can we exclude other factors that may influence the process of IVDD. In the current study, we do not determine whether elevated lipid levels directly give rise to LDH, or the elevation of lipids causes other disorders, which in turn lead to LDH. In order to prove cause and effect relationships and to find effective treatments for IVDD, a large longitudinal follow-up observations and intervention studies is needed [14].

In the present study, we excluded patients with diabetes, coronary heart disease, cerebrovascular disease associated with dyslipidaemia and focused only on patients with a pure single-level lumbar disc herniation to maximally reduce the risk of bias in our results. Other strengths of our study include the systematic collection of blood samples (TC, TG, HDL-C and LDL-C) and a preoperative assessment by imaging studies or a surgical diagnosis of herniated disc. Moreover, we are not aware of any study detailing the incidence of hyperlipidaemia or borderline hyperlipidaemia and the lipoprotein ratios in patients with a single-level lumbar disc herniation in the general Chinese adult population.

This study has certain limitations. First, we have no data on the concentrations of Apo A1, ApoB and Lp(a) in our patients. More detailed analysis could further reveal the the relationship between lipoprotein abnormalities and lumbar disc degeneration. Second, we only compared the herniated segment of the lumbar disc in the groups of patients with hyperlipidaemia and normal serum lipids, but we did not evaluate the degree of herniation. These parameters will be investigated in follow-up studies. In addition, power calculation was performed in the samples, and resulted in a relatively low statistical power (61.4 %), which may be due to the small sample size in this study. In the following investigations, we will enlarge the sample size to illustrate the potential association between serum lipids and disc herniation.

## Conclusions

In conclusion, the results of our study suggest that patients with higher LDL-C levels, High-TG and borderline High-LDL-C have a higher probability to developing IVDD. Serum lipid levels could be a useful predictor for IVDD in Chinese population. An enhanced understanding of this relationship may lead to new approaches for reducing the risk of IVDD.

## Methods

### Ethics statement

Ethical approval for this study was obtained from the Medical Ethics Committee of Shandong University and all participants provided written informed consent.

### Selection of subjects

The study included 790 subjects who were operated on at our institution (Qilu Hospital, Shandong University).

Group 1 (disc herniation) included 396 patients (235 men and 161 women; mean age: 41.07 years, range 18–79) who underwent surgery for single-level lumbar disc herniation (LDH) from January 2013 to December 2014 (Table 1). The levels of lumbar disc herniation were L1/2 in 1 patient, L2/3 in 4 patients, L3/4 in 7 patients, L4/5 in 186 patients, and L5/S1 in 198 patients.

Group 2 (control group) included 394 subjects (225 men and 169 women; mean age: 42.1 years, range 18–82) randomly selected from the patients who underwent surgery for wounded lower limbs in the same period [2, 45], and had no evidence of low back pain. The control and case groups were matched for age and gender (Table 1).

### Inclusion and exclusion criteria

Inclusion criteria for patients in group 1 were: (1) symptoms: low back pain with unilateral or bilateral lower limb radicular pain; (2) special nerve root irritation signs: straight leg raising test and strengthen test or femoral stretch test depending on the level; (3) neurologic deficit: muscle weakness, numbness, or lack of the corresponding reflex (knee jerk or ankle reflex); (4) computed tomographic (CT) or/and magnetic resonance imaging (MRI) with signs of a herniated disc. Exclusion criteria were: (1) lumbar spinal stenosis, spondylolisthesis, multiple intervertebral disc herniations, spinal tumour, history of spinal trauma and intervertebral space infection; (2) previous surgery on the affected lumbar disc.

Inclusion criteria for patients in group 2 were evidence of: lower limb fracture, a meniscal tear and cruciate ligament rupture diagnosed on the basis of clinical evaluation and imaging results. Exclusion criteria were: (1) history of spinal disorders, trauma and low back pain; (2) primary osteoarthritis of the operated or contralateral joint; (3) previous surgery on the affected lower limbs.

The common exclusion criteria for patients in group 1 and 2 were: (1) diabetes, coronary heart disease, cerebrovascular disease; (2) inflammatory arthritis; (3) patients younger than 18 years age.

### Collection of blood samples

Fasting blood samples were collected, and five millilitres of each fresh sample was centrifuged at 4,000 rpm for 6 min. Serum was extracted from the samples, and the concentrations of TC, TG, LDL-C and HDL-C were measured by an automatic biochemical analyser in an identical manner (Cobas 8000, Roche).

### Hierarchical criteria of lipid levels

According to the 2007 Chinese Guidelines on Prevention and Treatment of Dyslipidaemia in Adults, patients were considered to have: High-TC (hypercholesterolemia) if serum levels were ≥ 6.22 mmol/L and borderline high if levels were between 5.18 and 6.19 mmol/L; High-TG (hypertriglyceridemia) for levels ≥2.26 mmol/L and borderline high for levels between 1.70 and 2.25 mmol/L; High-LDL-C for levels ≥4.14 mmol/L and borderline high for levels between 3.37 and 4.12 mmol/L; Low-HDL-C for levels <1.04 mmol/L.

### Statistics

Continuous variables were expressed as the means ± standard deviations (SD) and analysed with independent-sample t-tests. Categorical variables were expressed as a percentage of the number and analysed with a chi-square test. Multivariate logistic regression was used to evaluate the effect of serum lipids on lumbar disc degeneration. Effect indicators were odds ratio (OR) and 95 % confidence interval (CI). SPSS (Version 17.0; Chicago, IL, USA) was used for all statistical analyses. All of the above tests were bilateral, and *P* < 0.05 was considered to be statistically significant. Additionally, power calculation was performed in both groups with PASS software.

## Abbreviations

BMI: body mass index
CT: computed tomographic
HDL-C: high-density lipoprotein cholesterol
IVD: intervertebral disc
IVDD: intervertebral disc degeneration
LBP: low back pain
LDH: lumbar disc herniation
LDL-C: low-density lipoprotein cholesterol
MRI: magnetic resonance imaging
TC: total cholesterol
TG: triglycerides

## References

[1] Andersson GB (1999). Epidemiological features of chronic low-back pain. Lancet.

[2] Jin G, Cao ZG, Zhang YN, Li Y, Shen BZ (2015). Physical activity is associated with elevated arterial stiffness in patients with lumbar disk herniation. J Spinal Disord Tech.

[3] Leino-Arjas P, Kauppila L, Kaila-Kangas L, Shiri R, Heistaro S, Heliovaara M (2008). Serum lipids in relation to sciatica among Finns. Atherosclerosis.

[4] Boos N, Semmer N, Elfering A, Schade V, Gal I, Zanetti M, Kissling R, Buchegger N, Hodler J, Main CJ (2000). Natural history of individuals with asymptomatic disc abnormalities in magnetic resonance imaging: predictors of low back pain-related medical consultation and work incapacity. Spine (Phila Pa 1976).

[5] Hirayama J, Yamagata M, Ogata S, Shimizu K, Ikeda Y, Takahashi K (2006). Relationship between low-back pain, muscle spasm and pressure pain thresholds in patients with lumbar disc herniation. Eur Spine J.

[6] Modic MT, Ross JS (2007). Lumbar degenerative disk disease. Radiology.

[7] Hussein AI, Jackman TM, Morgan SR, Barest GD, Morgan EF (2013). The intravertebral distribution of bone density: correspondence to intervertebral disc health and implications for vertebral strength. Osteoporos Int.

[8] Wade KR, Robertson PA, Thambyah A, Broom ND (2014). How healthy discs herniate: a biomechanical and microstructural study investigating the combined effects of compression rate and flexion. Spine (Phila Pa 1976).

[9] Austin MA, Hokanson JE, Edwards KL (1998). Hypertriglyceridemia as a cardiovascular risk factor. Am J Cardiol.

[10] Boullart AC, de Graaf J, Stalenhoef AF (1821). Serum triglycerides and risk of cardiovascular disease. Biochim Biophys Acta.

[11] Iatridis JC, MacLean JJ, Roughley PJ, Alini M (2006). Effects of mechanical loading on intervertebral disc metabolism in vivo. J Bone Joint Surg Am.

[12] Jamison D, Cannella M, Pierce EC, Marcolongo MS (2013). A comparison of the human lumbar intervertebral disc mechanical response to normal and impact loading conditions. J Biomech Eng.

[13] Takatalo J, Karppinen J, Taimela S, Niinimaki J, Laitinen J, Sequeiros RB, Samartzis D, Korpelainen R, Nayha S, Remes J, Tervonen O (2013). Association of abdominal obesity with lumbar disc degeneration--a magnetic resonance imaging study. PLoS ONE.

[14] Kauppila LI (2009). Atherosclerosis and disc degeneration/low-back pain--a systematic review. Eur J Vasc Endovasc Surg.

[15] Rastogi A, Kim H, Twomey JD, Hsieh AH (2013). MMP-2 mediates local degradation and remodeling of collagen by annulus fibrosus cells of the intervertebral disc. Arthritis Res Ther.

[16] Baillet A, Grange L, Trocme C, Caudroy S, Juvin R, Birembaut P, Morel F, Gaudin P (2013). Differences in MMPs and TIMP-1 expression between intervertebral disc and disc herniation. Joint Bone Spine.

[17] Vo NV, Hartman RA, Yurube T, Jacobs LJ, Sowa GA, Kang JD (2013). Expression and regulation of metalloproteinases and their inhibitors in intervertebral disc aging and degeneration. Spine J.

[18] Pockert AJ, Richardson SM, Le Maitre CL, Lyon M, Deakin JA, Buttle DJ, Freemont AJ, Hoyland JA (2009). Modified expression of the ADAMTS enzymes and tissue inhibitor of metalloproteinases 3 during human intervertebral disc degeneration. Arthritis Rheum.

[19] Dagistan Y, Cukur S, Dagistan E, Gezici AR. Importance of IL-6, MMP-1, IGF-1 and BAX Levels in Lumbar Herniated Discs and Posterior Longitudinal Ligament in Patients with Sciatic Pain. World Neurosurg. 2015. 10.1016/j.wneu.2015.07.039 26211852

[20] Studer RK, Vo N, Sowa G, Ondeck C, Kang J (2011). Human nucleus pulposus cells react to IL-6: independent actions and amplification of response to IL-1 and TNF-alpha. Spine (Phila Pa 1976).

[21] Singh K, Masuda K, Thonar EJ, An HS, Cs-Szabo G (2009). Age-related changes in the extracellular matrix of nucleus pulposus and anulus fibrosus of human intervertebral disc. Spine (Phila Pa 1976).

[22] Milgrom Y, Milgrom C, Constantini N, Applbaum Y, Radeva-Petrova D, Finestone AS (2013). The effect of very high versus very low sustained loading on the lower back and knees in middle life. Biomed Res Int.

[23] Sambrook PN, MacGregor AJ, Spector TD (1999). Genetic influences on cervical and lumbar disc degeneration: a magnetic resonance imaging study in twins. Arthritis Rheum.

[24] Abboud JA, Kim JS (2010). The effect of hypercholesterolemia on rotator cuff disease. Clin Orthop Relat Res.

[25] Ozgurtas T, Yildiz C, Serdar M, Atesalp S, Kutluay T (2003). Is high concentration of serum lipids a risk factor for Achilles tendon rupture?. Clin Chim Acta.

[26] Hemingway H, Shipley M, Stansfeld S, Shannon H, Frank J, Brunner E, Marmot M (1999). Are risk factors for atherothrombotic disease associated with back pain sickness absence? The Whitehall II Study. J Epidemiol Community Health.

[27] Leino-Arjas P, Kaila-Kangas L, Solovieva S, Riihimaki H, Kirjonen J, Reunanen A (2006). Serum lipids and low back pain: an association? A follow-up study of a working population sample. Spine (Phila Pa 1976).

[28] Hangai M, Kaneoka K, Kuno S, Hinotsu S, Sakane M, Mamizuka N, Sakai S, Ochiai N (2008). Factors associated with lumbar intervertebral disc degeneration in the elderly. Spine J.

[29] Longo UG, Denaro L, Spiezia F, Forriol F, Maffulli N, Denaro V (2011). Symptomatic disc herniation and serum lipid levels. Eur Spine J.

[30] Kauppila LI, Penttila A, Karhunen PJ, Lalu K, Hannikainen P (1994). Lumbar disc degeneration and atherosclerosis of the abdominal aorta. Spine (Phila Pa 1976).

[31] Kauppila LI, McAlindon T, Evans S, Wilson PW, Kiel D, Felson DT (1997). Disc degeneration/back pain and calcification of the abdominal aorta. A 25-year follow-up study in Framingham. Spine (Phila Pa 1976).

[32] Urban JP, Roberts S (2003). Degeneration of the intervertebral disc. Arthritis Res Ther.

[33] Kauppila LI (1997). Prevalence of stenotic changes in arteries supplying the lumbar spine. A postmortem angiographic study on 140 subjects. Ann Rheum Dis.

[34] Stairmand JW, Holm S, Urban JP (1991). Factors influencing oxygen concentration gradients in the intervertebral disc. A theoretical analysis. Spine (Phila Pa 1976).

[35] Steinberg D (2002). Atherogenesis in perspective: hypercholesterolemia and inflammation as partners in crime. Nat Med.

[36] Ebong IA, Goff DC, Rodriguez CJ, Chen H, Sibley CT, Bertoni AG (2013). Association of lipids with incident heart failure among adults with and without diabetes mellitus: Multiethnic Study of Atherosclerosis. Circ Heart Fail.

[37] Mendall MA, Patel P, Asante M, Ballam L, Morris J, Strachan DP, Camm AJ, Northfield TC (1997). Relation of serum cytokine concentrations to cardiovascular risk factors and coronary heart disease. Heart.

[38] Yoshida T, Kaneshi T, Shimabukuro T, Sunagawa M, Ohta T (2006). Serum C-reactive protein and its relation to cardiovascular risk factors and adipocytokines in Japanese children. J Clin Endocrinol Metab.

[39] Igarashi A, Kikuchi S, Konno S, Olmarker K (2004). Inflammatory cytokines released from the facet joint tissue in degenerative lumbar spinal disorders. Spine (Phila Pa 1976).

[40] Libby P (2002). Inflammation in atherosclerosis. Nature.

[41] Husain K, Hernandez W, Ansari RA, Ferder L (2015). Inflammation, oxidative stress and renin angiotensin system in atherosclerosis. World J Biol Chem.

[42] Mitra S, Goyal T, Mehta JL (2011). Oxidized LDL, LOX-1 and atherosclerosis. Cardiovasc Drugs Ther.

[43] Schaefer EJ, Asztalos BF (2006). The effects of statins on high-density lipoproteins. Curr Atheroscler Rep.

[44] Scicchitano P, Cameli M, Maiello M, Modesti PA, Muiesan ML, Novo S, Palmiero P, Saba PS, Pedrinelli R, Ciccone MM, Soc Italiana C (2014). Nutraceuticals and dyslipidaemia: Beyond the common therapeutics. J Funct Foods.

[45] Kurunlahti M, Tervonen O, Vanharanta H, Ilkko E, Suramo I (1999). Association of atherosclerosis with low back pain and the degree of disc degeneration. Spine (Phila Pa 1976).

